# Supplementary material for: A Global Screen for Assembly State Changes of the Mitotic Proteome by SEC-SWATH-MS
Source: Cell Syst. 2020 Feb 26;10(2):133–155.e6. doi: 10.1016/j.cels.2020.01.001 (PMC7042714; doi:10.1016/j.cels.2020.01.001)

Q7Z2W4 | ZCCHV_HUMAN | ZC3HAV1 ZC3HDC2 PRO1677
Monomer MW [kDa]: 101.431 Monomer expected elution fraction: 40

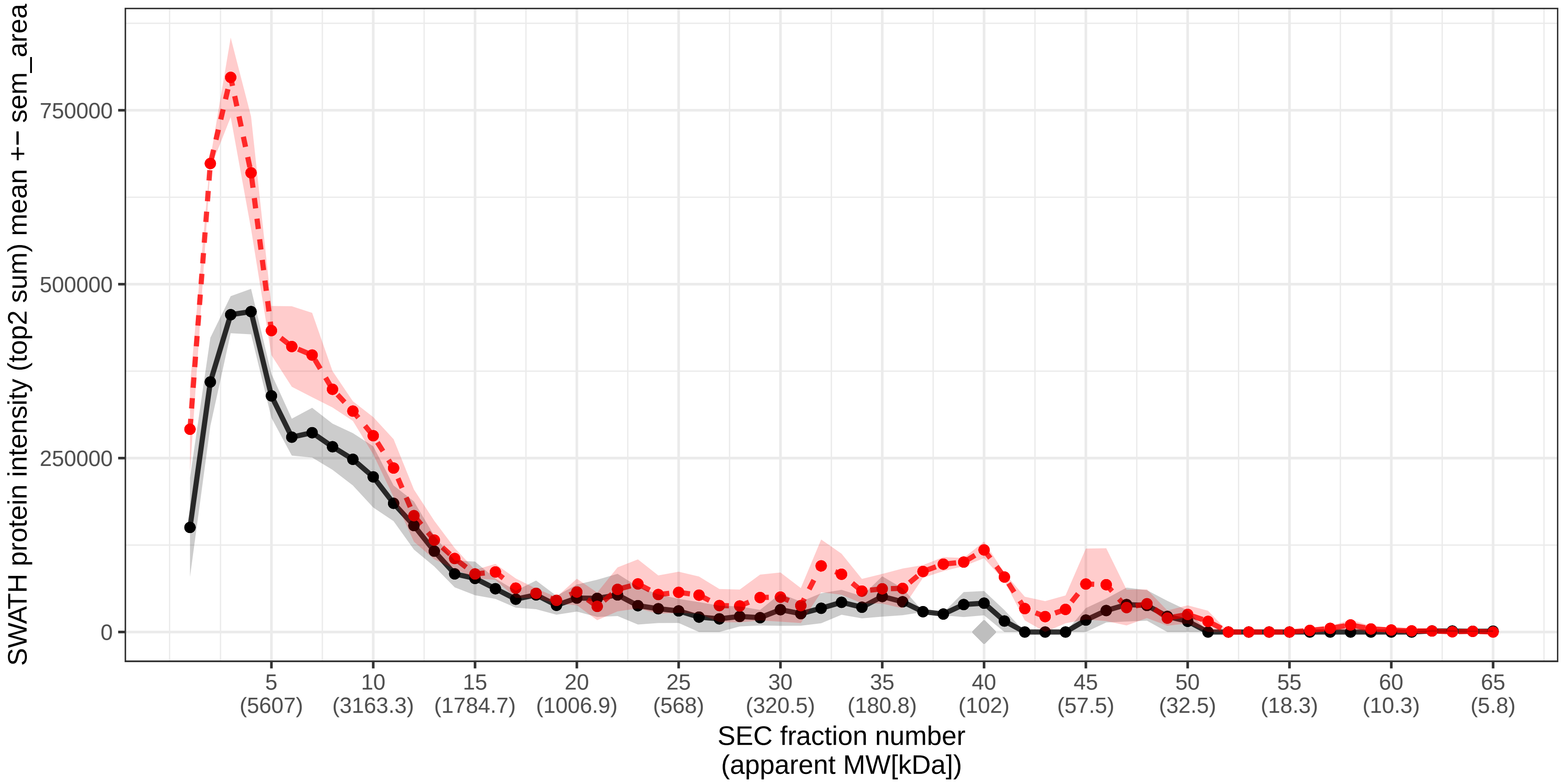

Supplement: Data S1. SEC-SWATH-MS Protein Chromatograms, Related to Figure 1 [file mmc6.zip › SECchrom_Q7Z2W4_ZCCHV_HUMAN_ZC3HAV1_ZC3HDC2_PRO1677.pdf]
